# The Utility of Immunohistochemistry in Differentiating Metastatic Primary Squamous Cell Carcinoma of the Thyroid from a Primary Lung Squamous Cell Carcinoma

**DOI:** 10.1155/2019/8641267

**Published:** 2019-10-17

**Authors:** Nern Hoong Kao, Chien Sheng Tan, Adrian Jit Hin Koh

**Affiliations:** ^1^Department of General Surgery, Changi General Hospital, 2 Simei Street 3, Singapore 529889; ^2^Department of Histopathology, Changi General Hospital, 2 Simei Street 3, Singapore 529889

## Abstract

Primary squamous cell carcinoma of the thyroid gland (PSCCTh) and anaplastic thyroid carcinoma with extensive squamous differentiation are rare entities which pose a diagnostic challenge in determining the primary site when presented as metastases. The difficulty in confirming a thyroid primary is further compounded by the aggressive nature of these tumours which frequently present at advanced stages. We present a case in which the patient presented with a thyroid mass and a lung mass simultaneously. The risk of misinterpreting the site of primary tumour as lung is greatly increased because squamous cell carcinoma of lung is much more common than its thyroid counterpart. This case highlights the effectiveness of PAX-8 stain in determining the primary site of tumour when squamous cell carcinoma is found in both lung and thyroid gland.

## 1. Introduction

Primary squamous cell carcinoma of the thyroid (PSCCTh) is a rare malignancy of the thyroid gland. According to Yang et al., PSCCTh accounts for only approximately 0.1% of all primary thyroid carcinomas [1]. A review of the SEER database yielded only 243 cases between the period of 1973–2015.

PSCCTh is an aggressive disease and holds a dismal prognosis with a median survival of six to nine months regardless of the modality of treatment received [1, 2]. However, patients with disease limited to the thyroid gland alone tend to have a better disease free survival [1, 3]. It is therefore imperative that PSCCTh be diagnosed early so as to initiate treatment as early as possible due to the aggressiveness of this disease.

Patients with PSCCTh commonly present with an enlarging anterior neck mass (60%). The patients may also develop dyspnea or dysphagia (20%) or with a change in their voice (15%). These patients tend to be after their 6^th^ decade of life and were more likely to be female [2]. Unfortunately, at the time of diagnosis, most patients were found with either locally advanced disease (76%), nodal (50%) or distant metastases (28%) [1].

Metastasis to the thyroid gland from lung squamous cell carcinoma is uncommon but likely to be more common than PSCCTh with lung metastases. The case presentation highlights the potential pitfall of misdiagnosing the lung tumour as a second primary, given the similarity between both tumours on morphology. In this context, PAX-8 immunohistochemical stain plays a vital role in determining the primary origin of squamous cell carcinoma.

## 2. Aim

Here, we present a rare case of metastatic PSCCTh masquerading as metastatic squamous cell carcinoma of the lung.

## 3. Case Presentation

A 69-year-old Chinese male presented via the emergency department with a one day history of mild shortness of breath. Upon closer interview, he also complained of a progressively enlarging left neck mass which had more than doubled in size over the period of six weeks. He did not note any stridor, hoarseness or dysphagia. The patient was a smoker of 50 pack years but did not consume alcohol. He had history of mild chronic obstructive pulmonary disease but otherwise did not have a family history of thyroid disorders. Physical examination showed a large (7 cm) left anterior neck mass which moved with swallowing. The mass was not tender and there was no retrosternal extension. There were no enlarged cervical lymph nodes or evidence of airway obstruction.

Blood tests performed showed a normal thyroid function with free thyroxine (fT4) at 12.85 pmol/L and thyroid-stimulating hormone at 0.619 *µ*IU/L. The white blood cell count (WBC) was also elevated at 21.6 × 10^9^/L. This was predominantly neutrophilic with an absolute neutrophil count of 17.9 × 10^9^/L. However, the other inflammatory markers were not significantly raised with a C-Reactive Protein (CRP) of 62 mg/L and a Pro-Calcitonin of 0.13 *µ*g/L.

**Figure 1:**
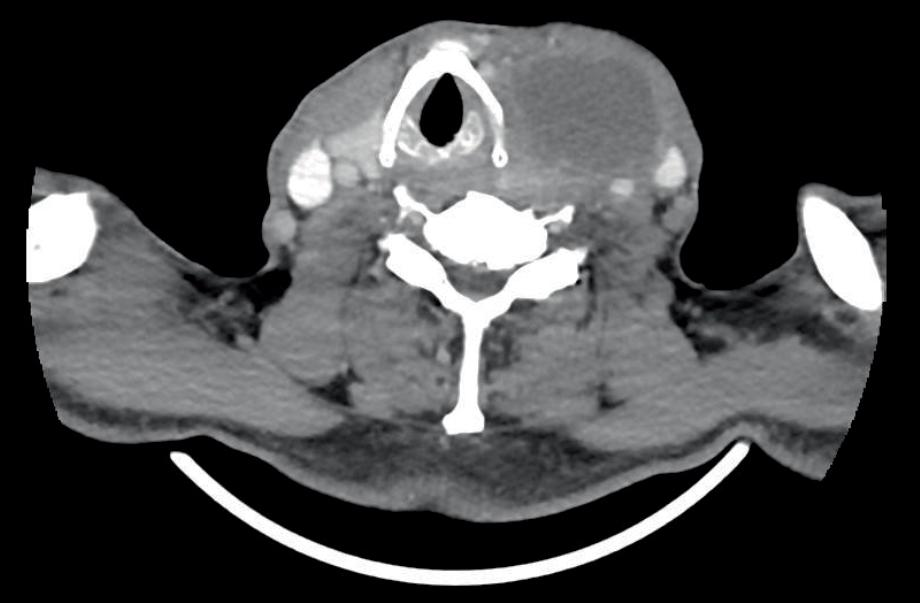
CT scan (axial) showing a large hypoechoic nodule with ill-defined nodular margins. This is suggestive of a solid-cystic nodule in the left thyroid.

**Figure 2:**
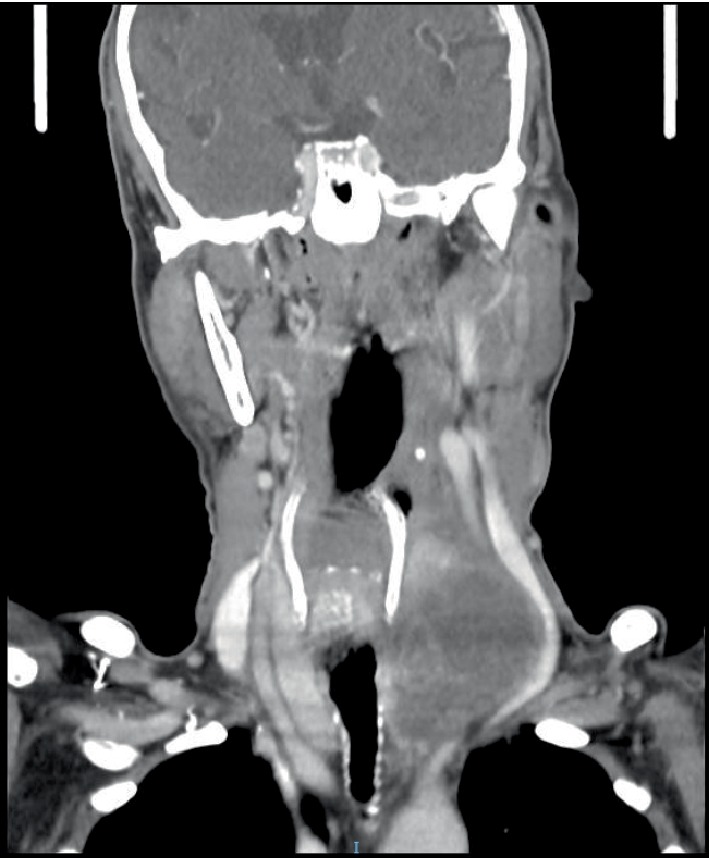
CT scan (coronal) showing gross deviation of the common carotid artery. The lack of fat plane suggests involvement of the carotid. The trachea is deviated but is not narrowed.

**Figure 3:**
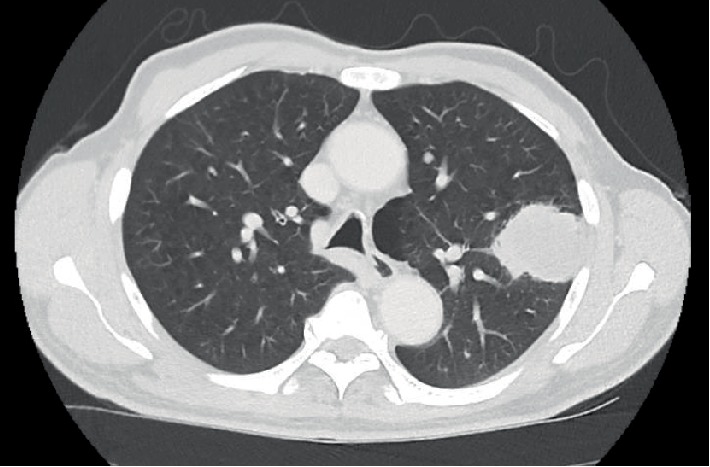
CT scan (axial) showing a 4.3 × 3.4 cm spiculated hyperechoic lesion in the left upper lobe abutting the pleura.

Contrast-enhanced CT scan (Figure 1) showed a 6.4 × 5.6 × 4.9 cm solid-cystic nodule arising from the left thyroid lobe and encasing the left common carotid artery. The trachea was deviated to the right but there was otherwise no airway narrowing (Figure 2). There were also several subcentimeter necrotic lymph nodes within the left cervical region. In addition, the CT scan also revealed a 4.3 × 3.4 cm spiculated mass in the left upper lobe of the lung as well as several other smaller subcentimeter lung nodules (Figure 3). A contrasted CT abdomen and pelvis did not show any intra-abdominal pathology. These radiological findings raised the possibility of synchronous tumours.

The patient underwent a bedside fine needle aspiration (FNAC) of which copious amount of pus was aspirated and concurred on cytology. In view of this finding, the authors were swayed towards the possibility of tuberculosis.

**Figure 4:**
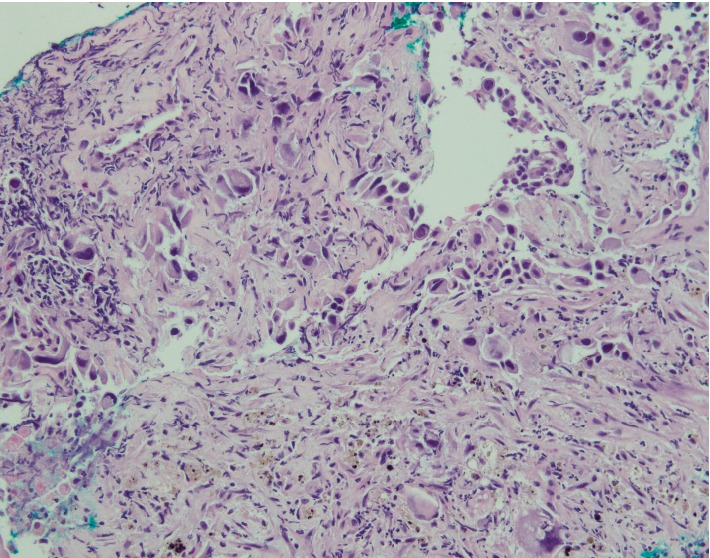
Lung parenchyma infiltrated by poorly differentiated carcinoma with squamoid features. Prominent background desmoplasia is noted.

An ultrasound-guided FNAC of the solid component of the nodule was arranged. This showed atypical squamous cells highly suspected for squamous cell carcinoma (Figure 4). No thyroid epithelial cells or anaplastic cells were seen in the cytology specimen. This finding contradicted the clinical impression of a common primary thyroid cancer. Acid fast bacilli (AFB) smears were negative.

**Figure 5:**
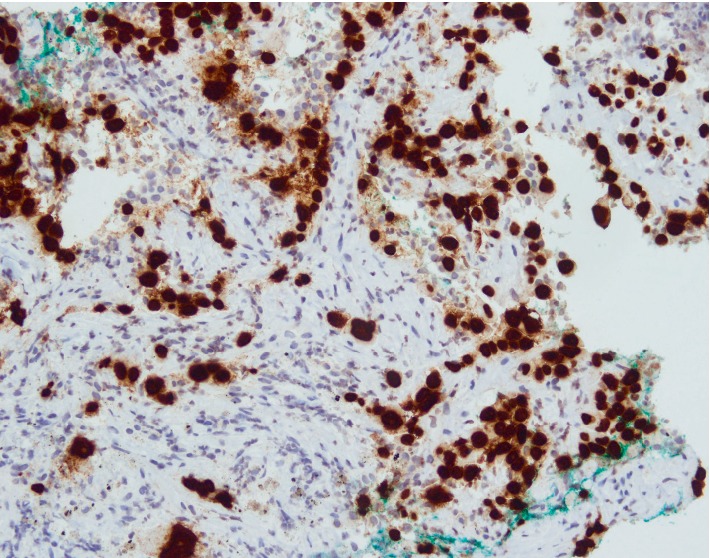
Tumour cells are diffusely immunoreactive to P40 stain. This finding is indicative of squamous differentiation. Diffuse P40 staining in tumour cells is in keeping with squamous cell carcinoma.

The patient also underwent a CT guided biopsy of the lung lesion. Histology showed poorly differentiated carcinoma with squamous differentiation. Immunohistochemistry staining (Figure 5) showed diffuse reactivity to p40, CK7 and paired box 8 (PAX-8) and were negative for Napsin A and thyroid transcription factor-1 (TTF-1). This was suggestive of a thyroid primary rather than a primary lung squamous cell cancer.

In view of this, the patient was diagnosed as primary squamous cell carcinoma of the thyroid with nodal and lung metastases. As most patients with advanced disease succumb to airway obstruction, the patient was offered an elective tracheostomy. However, he declined all treatment and opted for best supportive care. The patient passed away six weeks later.

## 4. Discussion

The clinical presentation with the rapid rate of growth was initially suggestive of anaplastic thyroid cancer. This was further conflicted by the presence of a large lung tumour and the presence of atypical squamous cells in the thyroid FNAC. The constellation of findings raised the possibility of a lung squamous cell carcinoma metastasizing to the thyroid gland or synchronous tumours. FNAC was not helpful in this case and it was not until tissue biopsy of the lung lesion was obtained that we could achieve the diagnosis. In a meta-analysis performed by Cho et al., FNAC had limited value in identifying PSCCTh. Only 27% of patients diagnosed with PSCCTh had prior FNAC results that showed SCC or suspicious for SCC.

**Figure 6:**
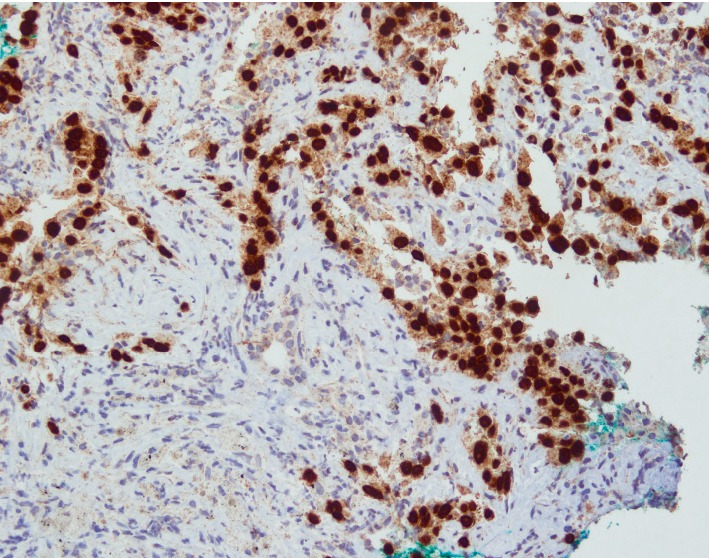
Tumour cells are also diffusely immunoreactive to PAX-8 stain. Coexpression of P40 and PAX-8 is supportive of a thyroid primary.

Tissue obtained from a biopsy or from a thyroidectomy specimen is more useful for diagnosis. The role of immunohistochemistry in assessing a metastatic tumour of unknown primary is well established. In this case, tissue obtained from the lung biopsy was invaluable as it stained positive for PAX-8 which was suggestive of a thyroid origin. It was unlikely to be a lung primary as the tissue was negative for Napsin A and TTF-1. Other microscopic features would include the presence of keratin or intercellular bridge structures within the thyroidal tissue [4, 5]. Suzuki et al. showed the rate of presence of PAX-8 to be as high as 91% in PSCCTh [6]. PAX-8 is a paired-box gene important in the embryogenesis of the thyroid gland, kidney, parathyroid gland, and Mullerian structures (Figure 6). It is markedly associated with thyroid gland organogenesis, thus the usefulness in identifying the most probable primary site in this case. Other useful markers include thyroglobulin (62.5%), p53 (50%), and TTF-1 (37.5%) [7].

One key point is the need to exclude local invasion of the thyroid from SCC of adjacent structures such as the larynx. This should be suspected should the specimen test negative for PAX-8. Imaging such as CT scans are a useful adjunct for this purpose. In a systematic review by Syed et al., there were no specific radiological features to aid in the diagnosis of PSCCTh. However, CT scans are useful to exclude other potential sources of SCC with thyroid metastases or causing direct invasion of the thyroid gland. PSCCTh was also found to be more likely to encase the oesophagus rather than invade into the oesophagus. This is helpful in excluding an oesophageal primary invading into the thyroid as there would still be a fat plane seen around the oesophagus. CT scans were also found to be useful in assessing the severity of airway compression [8].

The success of treatment largely depends on being able to achieve R0 resection. Disease free survival is further improved if adjuvant radiotherapy or radiochemotherapy is given [9, 10]. However, radiotherapy and/or chemotherapy alone without surgical treatment holds a dismal outcome as PSCCTh is relatively radioresistant [7, 11]. Multiple studies have also shown PSCCTh to be poorly responsive to chemotherapy [8]. PSCCTh is similar to anaplastic thyroid carcinoma and does not uptake iodine. There is no role for radioactive iodine ablation or thyroid suppression. Recent development in the area of immunotherapy based on programmed cell death ligand 1 inhibitors (PD-L1) might prove to be a viable option for a small number of patients with tumour showing high expression of PD-L1 on immunohistochemistry studies [12].

## 5. Conclusion

Primary squamous cell carcinoma of the thyroid gland with a dominant metastatic tumour could pose a diagnostic challenge due to its rarity and indistinguishable morphological features from other primary squamous cell carcinomas. In such clinical settings, PAX-8 immunohistochemical stain could prove to be useful in determining the primary origin of squamous cell carcinoma.
